# EHMTI-0232. Non-surgical management of rhinogenic contact point headache

**DOI:** 10.1186/1129-2377-15-S1-C1

**Published:** 2014-09-18

**Authors:** N Ahmady Roozbahany, S Nasri

**Affiliations:** 1 Otolaryngology, Shahid Beheshti Medical University, Tehran, Iran; 2 Public Health, Private Practice, Tehran, Iran

## Introduction

Rhinogenic contact point headache (RCPH) is a pain that arises from two opposing mucosal surfaces in the nose. The diagnosis is made by history and physical examination along with nasal endoscopy and imaging. The mainstay of treatment is surgical removal of contact area.

## Aims

To evaluate the effectiveness of non-surgical managements including behavioral and pharmacotherapy in treatment of RCPH.

## Methods

Thirty patients with confirmed diagnosis of RCPH underwent non-surgical management of RCPH using recommendations for absolute contralateral nostril breathing during the pain period, applying warm cushion and medical treatment with non-steroidal analgesics and beta blocker. The results of treatment compared with the results of a group of patients who underwent surgical management.

## Results

More than eighty six percent of patients experienced a significant relief in the severity of the pain. In contrast to the surgically managed group, the frequency of headaches was not altered.

## Conclusion

Non-surgical management of RCPH may have a role in patients who do not accept surgery or are not a candidate for surgical management.

No conflict of interest.

